# The Effect of Asfotase Alfa on Plasma and Urine Pyrophosphate Levels and Pseudofractures in a Patient With Adult‐Onset Hypophosphatasia

**DOI:** 10.1002/jbm4.10842

**Published:** 2023-11-20

**Authors:** Naoko Hidaka, Hiroaki Murata, Kanako Tachikawa, Keiichi Osaki, Takashi Sekiyama, Yuka Kinoshita, Hajime Kato, Yoshitomo Hoshino, Soichiro Kimura, Takashi Sunouchi, So Watanabe, Masaomi Nangaku, Noriko Makita, Toshimi Michigami, Nobuaki Ito

**Affiliations:** ^1^ Division of Nephrology and Endocrinology The University of Tokyo Hospital Tokyo Japan; ^2^ Osteoporosis Center The University of Tokyo Hospital Tokyo Japan; ^3^ Department of Orthopaedic Surgery, Panasonic Health Insurance Organization Matsushita Memorial Hospital Osaka Japan; ^4^ Department of Bone and Mineral Research, Research Institute Osaka Women's and Children's Hospital Osaka Japan; ^5^ Department of Rehabilitation, Panasonic Health Insurance Organization Matsushita Memorial Hospital Osaka Japan; ^6^ Department of Geriatric Medicine, Graduate School of Medicine The University of Tokyo Tokyo Japan

**Keywords:** DISORDERS OF CALCIUM/PHOSPHATE METABOLISM (OTHER), HUMAN ASSOCIATION STUDIES, INJURY/FRACTURE HEALING, OSTEOMALACIA AND RICKETS, THERAPEUTICS (OTHER)

## Abstract

Hypophosphatasia (HPP) is an inherited disease caused by variants of the *ALPL* gene encoding tissue‐nonspecific alkaline phosphatase. Adult‐onset HPP (adult HPP), known as a mild form of HPP, develops symptoms involving osteomalacia after the age of 18 years. Asfotase alfa (AA) is a modulated recombinant human alkaline phosphatase (ALP) that has been established as a first‐line therapy for severe forms of HPP, such as perinatal and infantile forms. We described a 64‐year‐old female who presented with pseudofractures in bilateral femur diaphyses and impaired mobility. Low serum ALP activity and a high concentration of urine phosphoethanolamine indicated the diagnosis of HPP, which was confirmed by the identification of a homozygous variant in the *ALPL* gene (c.319G > A; p.Val107Ile). An in vitro transfection experiment to measure the ALP activity of this novel variant protein was performed, resulting in 40% of the residual enzymatic activity compared with the wild type. AA was initiated to facilitate the union of pseudofracture and to improve mobility. After 6 months, radiographic images revealed the disappearance of fracture lines, and improvement of ambulatory ability was confirmed by the 6‐minute walk test (525 to 606 m). The EQ‐5D‐5L index was also improved (0.757 to 0.895). Within a follow‐up period, the levels of urine pyrophosphate corrected by urine creatinine (uPPi/Cre) declined in parallel with the level of plasma PPi (plasma PPi: 6.34 to 1.04 μM, uPPi/Cre: 226.8 to 75.4 nmol/mg). The beneficial effect of AA on pseudofracture healing in adult HPP was presented, although the application of AA should be restricted to patients exhibiting relatively severe manifestations. In addition, a novel pathogenic variant of the *ALPL* gene was identified with the supportive result of functional analysis. Furthermore, when monitoring patients with HPP treated with AA, uPPi/Cre might be a convenient substitute for plasma PPi, which requires immediate filtration after blood sampling. © 2023 The Authors. *JBMR Plus* published by Wiley Periodicals LLC. on behalf of American Society for Bone and Mineral Research.

## Introduction

Hypophosphatasia (HPP) is a congenital bone metabolic disease caused by pathogenic variants in the *ALPL* gene encoding tissue‐nonspecific alkaline phosphatase (TNSALP). Reduced enzymatic activity of TNSALP decreases the decomposition of inorganic pyrophosphate (PPi) into inorganic phosphate (Pi), which induces defective mineralization of bone, resulting in osteomalacia/rickets and increasing the risk for developing calcium pyrophosphate deposition (CPPD) disease. HPP is categorized into six forms by clinical characteristics and the age of onset: perinatal, benign prenatal, infantile, childhood, adult, and odontohypophosphatasia. Adult‐onset HPP (adult HPP) develops in patients after 18 years of age and is typically diagnosed in middle age with heterozygous variants of *ALPL* with dominant negative effects or compound heterozygous variants.^[^ ^1^, ^2^ ^]^ Clinical characteristics are greatly variable among patients with adult HPP, from patients with disabling features, including multiple fractures, chondrocalcinosis, and dental abnormalities, to almost asymptomatic patients with nonspecific manifestations, such as chronic muscle weakness and pain, joint pain probably due to CPPD disease, headache, and fatigue.^[^ ^3^, ^4^ ^]^

Asfotase alfa (AA) (Alexion Pharmaceuticals, Inc., Boston, MA, USA), which is a modulated human recombinant alkaline phosphatase (moduALP) with high affinity to hydroxyapatite, is a drastic treatment for HPP.^[^ ^5^ ^]^ AA has been shown to improve skeletal mineralization, respiratory function, growth, and cognitive and motor function in patients with perinatal or infantile HPP until 7 years of follow‐up.^[^ ^6^, ^7^, ^8^ ^]^ AA is now established as a first‐line therapy for these severe forms of HPP; however, in European countries and the United States, administration for adults is approved only for adult patients with pediatric‐onset HPP, not for adults with adult‐onset HPP. In contrast, the use of AA among adult HPP regardless of onset age is legitimate in Japan, although the selection of patients requires discretion in terms of cost‐effectiveness. Thus, appropriate selection of the patients and cautious monitoring to assess efficacy and safety are recommended when physicians initiate AA for adult HPP.^[^ ^9^ ^]^

We previously reported that radiographic studies, including bone scintigraphy, aided in monitoring the improvement of pseudofracture in a patient with prenatal benign HPP under treatment with AA.^[^ ^10^ ^]^ Although imaging studies (X‐ray, magnetic resonance imaging [MRI], bone scintigraphy, etc.) and assessments of mobility, musculoskeletal function (6‐minute walk test, muscle strength, gait, etc.), and quality of life (QOL; EQ‐5D‐5L, SF‐36, etc.) are recommended for monitoring the effect of AA, laboratory examinations are also important.^[^ ^11^ ^]^ To date, measurable parameters for the monitoring of HPP patients treated with AA include plasma pyridoxal‐5′‐phosphate (PLP), plasma PPi, and urinary phosphoethanolamine (uPEA). Among them, high PPi levels are assumed to be an inhibitor of hydroxyapatite formation and bone mineralization, and high PPi levels are also supposed to be associated with the development of CPPD disease.^[^ ^12^, ^13^ ^]^ Therefore, plasma PPi levels would be a good marker for the efficacy of AA among measurable markers. In Japan, measurement of uPEA is commercially available, while plasma PLP and PPi could be measured in limited institutions under the research settings.

In this report, a patient with adult HPP who experienced bilateral subtrochanteric fragility fractures and impaired mobility was presented. AA was initiated to facilitate fracture healing and improve the patient's mobility. A detailed presentation of the current patient and the changes in laboratory parameters and imaging in response to AA are described below.

## Case Description

**Fig. 1:**
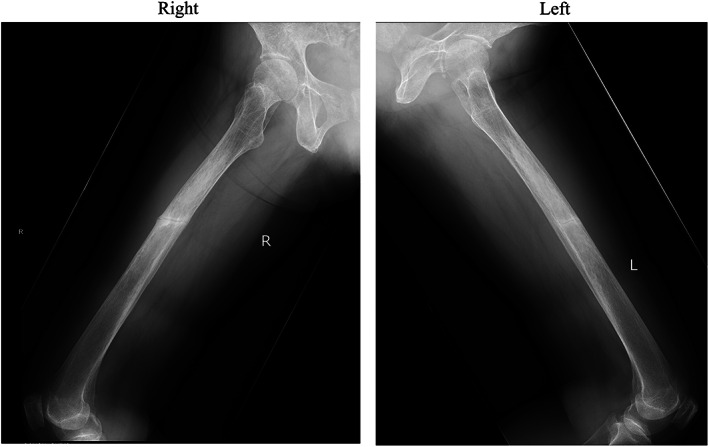
X‐rays of the femur at the initial visit.

**Fig. 2:**
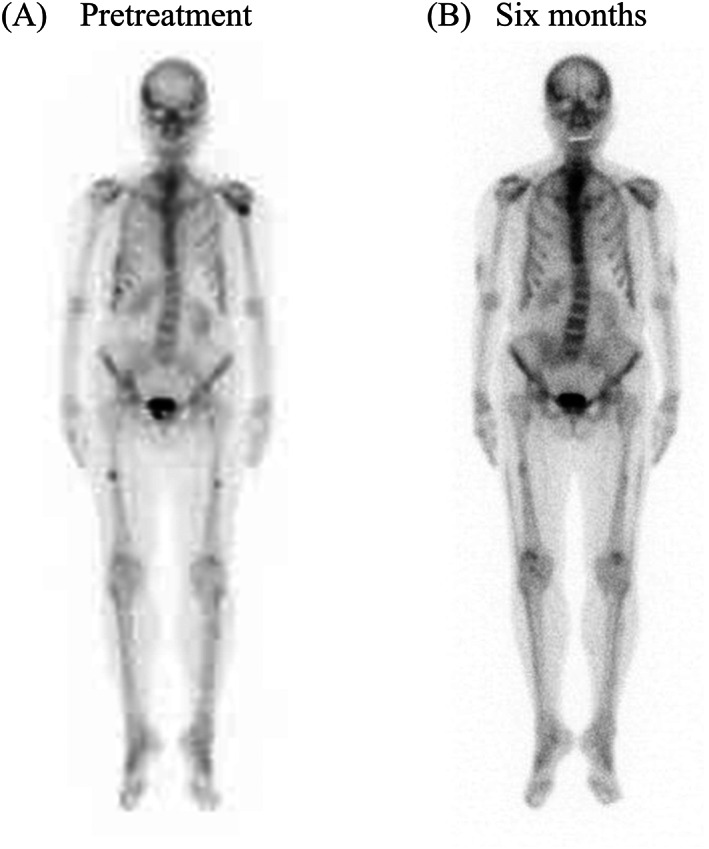
Bone scintigraphy (*A*) at the initial visit and (*B*) at 6 months after the initiation of asfotase alfa.

A 64‐year‐old woman who had an injection of romosozumab (210 mg/mo) for the treatment of osteoporosis for 6 months came to the orthopedic clinic with a chief complaint of right femoral pain that was exacerbated while walking. X‐ray and MRI showed pseudofractures in the diaphyses of the bilateral femur, suggestive of metastasis of malignancy or some bone metabolic disorders other than osteoporosis (Fig. 1). She had no history of fracture or pseudofracture until she had a fracture in the left humerus at age 62 years, which led to the diagnosis of osteoporosis. Three weeks later, she was introduced to the orthopedic department of our hospital for further examination. On the initial visit, she presented with difficulties running and rising from a squat. She had no history of glucocorticoid or bone antiresorptive agent administration, including bisphosphonate. She had lost all teeth at age 40 years, while it was not clear when her deciduous teeth completely fell out. Her parents had a cousin marriage, and her mother and elder sister were prone to fracture. She had two daughters and a son who had no history of bone disorders. Laboratory tests revealed low levels of serum alkaline phosphatase and high levels of serum albumin‐corrected calcium (Ca), Pi, and uPEA, all of which indicated adult HPP (Table 1). The bone mineral density (BMD) of the left femoral neck was low (Table 1). Bone scintigraphy revealed multiple hot spots in the left proximal humerus, ribs, and diaphyses of the bilateral femur (Fig. 2*A*). A CT scan of the whole body did not indicate any tumors or ectopic ossifications. A genetic test for the *ALPL* gene was conducted to confirm the diagnosis of adult HPP.

**Table 1:** Laboratory Data, Bone Mineral Density, Physical Tests, and Quality of Life Assessment Before and 6 Months After the Initiation of Asfotase Alfa

	Reference range	Pretreatment	Six months
Laboratory data			
Alkaline phosphatase (IU/L)	38–113	8	13,336
Bone alkaline phosphatase (μg/L)	3.8–22.6	1.2	3130
TRACP‐5b (mU/dL)	120–420	476	1500
Serum albumin‐corrected calcium (mg/dL)	8.2–9.8	9.9	10.1
Serum inorganic phosphate (mg/dL)	2.3–4.3	5.4	6.0
Intact parathyroid hormone (pg/mL)	10–65	43	60
25‐hydroxyvitamin D (ng/mL)	30.0‐	25.4	16.1
Urinary phosphoethanolamine (μmol/gCr)	31–110	545	97
Bone mineral density (*Z*‐score)			
Lumbar (L_2_ to L_4_)		0.0	0.3
Left femoral neck		−2.2	−2.1
Physical tests			
Hand‐grip strength (kg; right/left)		23.2/22.5	23.0/20.6
6‐minute walk test (m)		525	606
Timed up‐and‐go test (s)		6.92	6.17
Quality of life assessment			
EQ‐5D‐5L index^a^		0.757	0.895
Mobility		2	1
Self‐care		1	1
Usual activities		2	1
Pain, discomfort		3	2
Anxiety, depression		1	1

^a^ The mean (standard deviation) value of the EQ‐5D‐5L index of females aged 60–69 years established in the Japanese population is 0.927 (0.104).^[^ ^30^ ^]^

## Materials and Methods

### Genetic test for the *ALPL* gene

Genomic DNA was extracted from peripheral blood leukocytes and subjected to polymerase chain reaction (PCR) to amplify the sequences corresponding to each coding exon of *ALPL* with exon/intron boundaries. The PCR products were gel‐purified and directly sequenced by Sanger sequencing.

### Functional analysis of ALP protein encoded by the *ALPL* gene with the identified variant

Functional analysis of the identified *ALPL* variant in the current patient was first conducted by in silico tools, including SIFT (http://sift-dna.org/sift4g), Polyphen‐2 (http://genetics.bwh.harvard.edu/pph2/), CADD (https://cadd.gs.washington.edu/), and MutationTaster2021 (https://www.genecascade.org/MutationTaster2021/).

Next, using ALP cDNA, the expression plasmid for green fluorescent protein (GFP)‐tagged TNSALP (pcDNA‐GFP‐ALP) was constructed,^[^ ^14^ ^]^ and the identified *ALPL* variant (c.319G > A; p.Val107Ile) was introduced with the QuikChange Lightning Site‐Directed Mutagenesis Kit (Agilent Technologies, Santa Clara, CA, USA). For comparison, the expression plasmid for GFP‐tagged mutant TNSALP (c.1559delT; p.Leu520ArgfsX86), which is the most prevalent loss‐of‐function variant in the Japanese population, was prepared. The expression plasmids of the wild type, two variant TNSALP (p.Val107Ile identified in the current case and p.Leu520fs), and vitamin D receptor (pSG5‐VDR‐GFP) as a negative control were introduced into COS‐7 cells using FuGENE HD Reagent (Roche, Indianapolis, IN, USA). Three days after transfection, cell lysates were harvested in 10 mM Tris‐Cl and 0.05% Tris‐Triton X‐100. The enzymatic activity of ALP in the cell lysates was measured using p‐nitrophenylphosphate as a substrate. The expression of GFP‐tagged protein (TNSALP or VDR) was confirmed by Western blotting using aliquots of the cell lysates and the anti‐GFP antibody. Densitometry of the signals was performed using ImageJ software, and the enzymatic activity of the cell lysates was normalized based on the signal intensity.

### PPi measurement

At 0, 2, and 7 days, 2 weeks, and 1, 2, 4, 5, and 6 months after the initiation of AA, blood samples were collected into heparin‐treated tubes and immediately placed on ice. By centrifugation at 2200 *g* for 5 minutes, 400 μL of blood plasma was isolated and collected into a centrifugal tube with a 300 kDa membrane (Nanosep centrifugal filter, Pall Life Sciences, Portsmouth, UK). The filtered tube was centrifuged at 14,000*g* for 20 minutes, and aliquots of the obtained flowthrough were stored frozen at −80°C. The sequential procedure was performed at 4°C within 1 hour. Urine samples were collected at the same time as the plasma collection and then stored frozen at −80°C. The concentration of PPi was measured by the ATP sulfurylase method as previously described with minor modifications.^[^ ^15^, ^16^ ^]^ The luminescence signal was measured by an EnSpire multimode plate reader (PerkinElmer, Waltham, MA, USA) at room temperature. PPi concentrations were calculated from the calibration curve. The concentration of each sample was presented as the mean of the concentrations calculated from double aliquots. The urine PPi concentration was corrected by the urine creatinine concentration of the same sample. The samples with PPi concentrations above 5 μM were diluted 1:10–100 with ATP‐free water and then assayed again. The reference range of PPi in healthy children and adolescents measured by the ATP sulfurylase method was previously reported as 2.36 to 4.44 μM, which was similar to the standard ranges measured by the uridine‐diphosphoglucose pyrophosphorylase method in healthy adults.^[^ ^17^, ^18^ ^]^ The data were analyzed using R software version 4.3.0.

### Ethics approval

The current study was approved by the institutional review board of the University of Tokyo Hospital (approval numbers: 11221, G10115, and 2879). Written informed consent of the participant was obtained before the study.

## Results

### Variant of the *ALPL* gene and functional analysis of the identified variant ALP

**Fig. 3:**
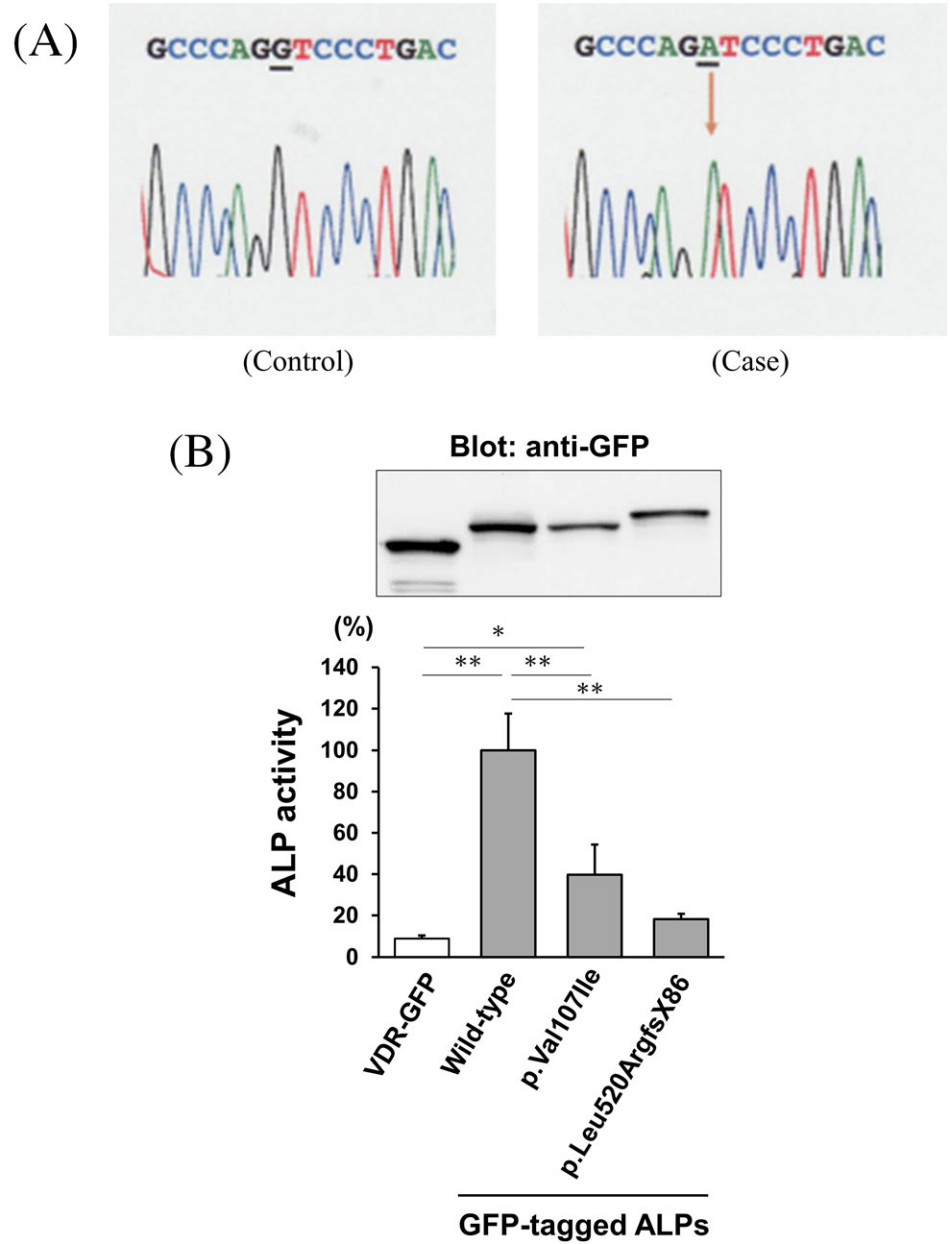
(*A*) The novel variant of the *ALPL* gene in the current case. Sanger sequencing identified a homozygous missense variant in exon 5 of the *ALPL* gene (c.319G > A; p.Val107Ile). (*B*) The bar graph shows the mean with standard deviation of the enzymatic activity. The p.Val107Ile variant of TNSALP was decreased to approximately 40% of the wild type. As a negative control, a fusion protein of vitamin D receptor and GFP (VDR‐GFP) was introduced. For comparison, the p.Leu520ArgfsX86 (c.1559delT) variant of TNSALP, which is the most prevalent variant in the Japanese population, was coassayed. The enzymatic activity was normalized based on the signal intensity in Western blotting using aliquots of the cell lysates and an anti‐GFP antibody. The enzymatic activity in the cells expressing wild‐type TNSALP was designated as 100%. Data are shown as the mean ± SD (*n* = 3). *p < 0.05, **p < 0.01 using one‐way ANOVA with the Tukey–Kramer test.

Genetic testing identified a homozygous missense variant in exon 5 of *ALPL* (c.319G > A; p.Val107Ile) (Fig. 3*A*), which was novel, and in silico analysis of this variant predicted it to be tolerant (SIFT) or damaging (Polyphen‐2, CADD, and MutationTaster2021) (Supplemental Table S1).

To confirm the deleterious effect of this novel *ALPL* variant, functional analysis by in vitro transfection was also performed. The ALP activity of cells expressing the current *ALPL* variant (c.319G > A; p.Val107Ile) was approximately 40% of those expressing the wild type, which confirmed the diagnosis of adult HPP. The loss of enzymatic activity in this variant was estimated to be milder than that of the most prevalent pathogenic variant in Japanese individuals (c.1559delT; p.Leu520ArgfsX86), which showed almost complete loss of ALP activity (Fig. 3*B*).

### Treatment and changes in imaging tests

**Fig. 4:**
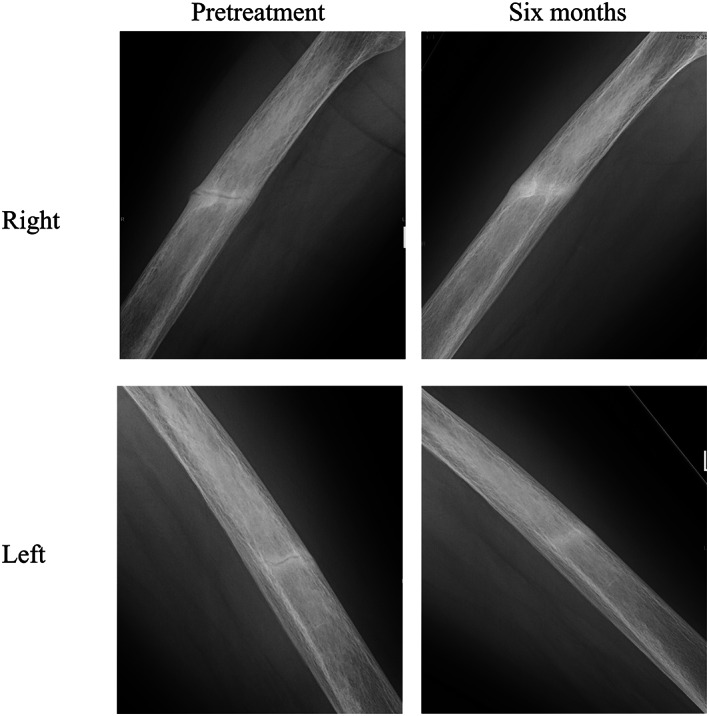
Radiographic improvement of the pseudofractures observed in bilateral femoral diaphyses, 6 months after the initiation of asfotase alfa.

The results of the in vitro functional analysis and her symptoms, including early loss of her permanent teeth and incomplete fractures in the diaphyses of bilateral femurs, which are typical of HPP, led to the diagnosis of adult HPP, and AA (1.7 mg/kg, 3 times/wk) was initiated. Six months after the initiation of AA, her mobility improved with the disappearance of general pain, which contributed to the improvement in the EQ‐5D‐5L index. The results of the 6‐minute walk test and timed up‐and‐go test also improved (Table 1). The levels of serum ALP and bone alkaline phosphatase (BAP) reached extremely high levels, reflecting the successful injection of AA, and the level of uPEA decreased into the reference range in response to AA treatment (Table 1). The levels of serum albumin‐corrected Ca and Pi remained high (Supplemental Fig. S1; Table 1). X‐ray of the bilateral femoral diaphyses revealed the disappearance of fracture lines after 6 months (Fig. 4). In bone scintigraphy, multiple hot spots suggesting pseudofractures were observed, especially in the ribs, bilateral femurs, and left humerus, which diminished after 6 months (Fig. 2*B*), whereas BMD did not exhibit a significant change (Table 1). The patient experienced mild infusion‐site reactions, including pain, swelling, and internal bleeding. The accumulation of nuclides in the upper arms and thighs observed by bone scintigraphy was suspected to indicate the inflammation of soft tissues due to AA injection (Fig. 2*B*).

### Changes in serum and urine PPi

**Fig. 5:**
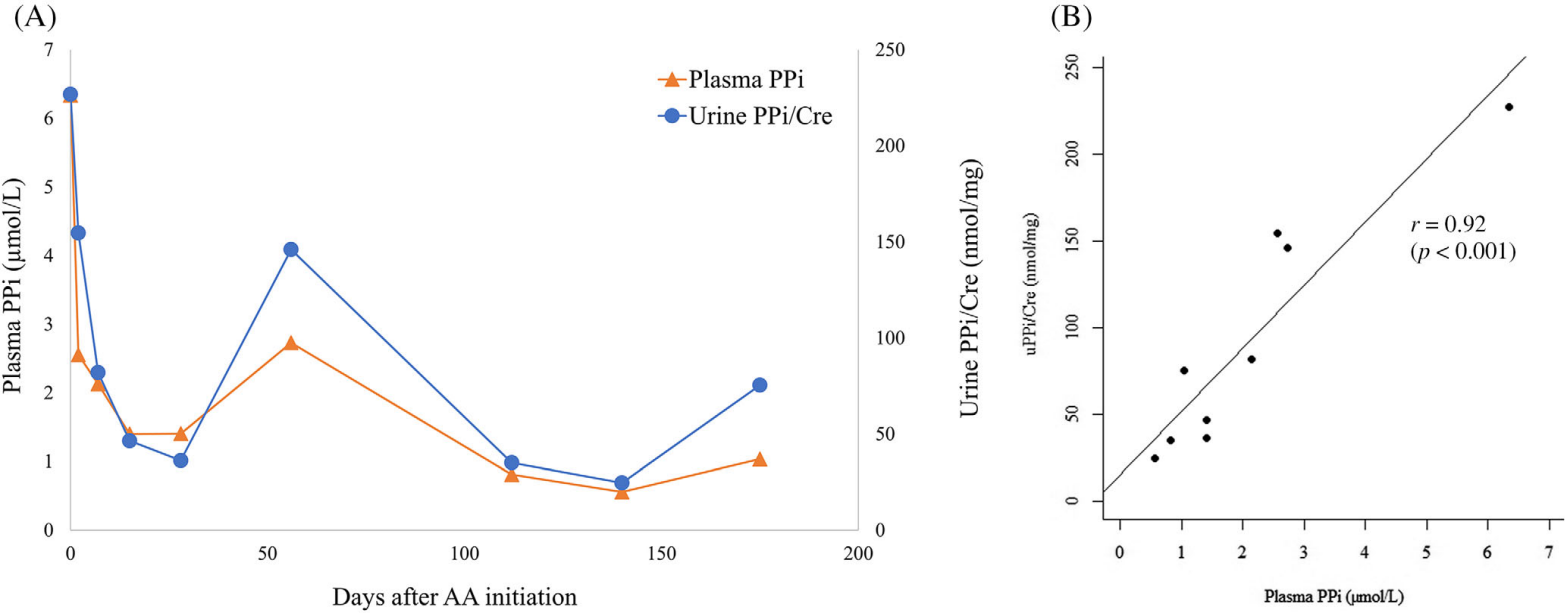
(*A*) Changes in plasma and urine pyrophosphate (PPi) concentrations after the initiation of asfotase alfa (AA). Urine PPi was corrected by urinary creatinine (Cre) concentration at each date. (*B*) Correlation between plasma PPi and uPPi/Cre. Pearson's correlation coefficient was 0.92 (*p* < 0.001).

The level of plasma PPi before treatment was 6.34 μM, which was above the reference range (2.36–4.44 μM),^[^ ^17^, ^18^ ^]^ which was compatible with the diagnosis of HPP. After the initiation of AA, the level of plasma PPi immediately decreased to the reference range within a week. Urine PPi corrected by urine creatinine (uPPi/Cre) also showed a decrease in parallel to plasma PPi. The levels of plasma PPi remained normal or low throughout the 6‐month observation period (plasma PPi: 6.34 to 1.04 μM, uPPi/Cre: 226.8 to 75.4 nmol/mg) (Fig. 5*A*). The uPPi/Cre level was highly correlated with the plasma PPi level (*r* = 0.92, *p* < 0.001, Fig. 5*B*).

## Discussion

This report presented a case of adult HPP that was successfully treated by AA. Based on a serial measurement of plasma and urine PPi within 6 months after the initiation of AA, we demonstrated that the level of uPPi/Cre decreased in line with plasma PPi after the initiation of AA and that plasma PPi and uPPi/Cre levels were highly correlated with each other throughout 6 months (Fig. 5*A*, *B*). To measure plasma PPi levels, plasma samples need to be filtered soon after the collection of blood because leakage of PPi from blood cells, especially platelets, interferes with obtaining accurate measurements. Compared with plasma samples, urine samples for PPi measurement do not require this filtering step. After the initiation of AA, the serum level of ALP activity is extremely elevated. Although monitoring ALP activity might be considered to assess adherence to AA treatment, the significance is undetermined when estimating the efficacy of AA in the bone.^[^ ^11^ ^]^ Therefore, the measurement of PPi, which is the substrate of ALP and is also known as an inhibitor of mineralization in bone, should be underscored in monitoring the efficacy of AA. Although there is no guarantee of this precise correlation between uPPi/Cre and plasma PPi in other patients with HPP, uPPi/Cre may be a more feasible biomarker than plasma PPi in the real world, which would aid in monitoring an HPP patient under enzyme replacement therapy. Moreover, it would also be a convenient diagnostic aid for HPP and disorders with decreased plasma PPi levels, such as generalized arterial calcification of infancy, autosomal recessive hypophosphatemic rickets 2, and pseudoxanthoma elasticum with *ENPP1* and *ABCC6* variants.^[^ ^15^, ^19^, ^20^, ^21^ ^]^

Contrary to the result in our case, a previous report on a murine model of HPP (*ALPL* ^−/−^ mice) injected with adeno‐associated virus (AAV) 8‐TNSALP‐deca‐aspartate (D_10_) did not present uPPi/Cre reduction after treatment with AAV8‐TNSALP‐D_10_, although plasma PPi significantly decreased.^[^ ^22^ ^]^ AAV8‐TNSALP‐D_10_ differs from AA in that the latter has a human IgG1 Fc domain for drug purification; however, they are qualitatively identical treatments. Although it is difficult to clearly explain the difference in the uPPi/Cre reaction to AA and AAV8‐TNSALP‐D_10_, most uPPi is known to be derived from the kidney^[^ ^23^ ^]^; thus, the different reaction of uPPi/Cre to these two treatments might underlie the effectiveness of these two treatments in the kidney. Future research to focus on the effect of AA in extraskeletal organs, including the kidney, is warranted, in addition to surveillance of the range of uPPi/Cre in healthy participants and the correlation between uPPi/Cre and plasma PPi among other pediatric and adult patients with HPP treated with AA.

There have been few case reports of adult‐onset HPP treated with AA, as its use is not permitted in European countries and the United States for these cases.^[^ ^24^, ^25^ ^]^ Clinical manifestations of adult HPP vary in patients and include nonspecific symptoms such as joint pain and headache, and discrimination of actual HPP‐related symptoms is often difficult. Although there are no criteria for initiating AA in adult HPP in Japan, where the use of AA is also approved among adult HPP, physicians should select appropriate patients who truly need treatment with AA. Furthermore, when initiating AA in adult HPP, treatment goals and monitoring parameters must be predetermined to avoid unnecessary overtreatment and clinical inertia. In the current report, we monitored laboratory tests, imaging studies (X‐ray and bone scintigraphy), physical assessments, and QOL scoring. The chief goal of the treatment was healing of the fractures, while improving physical ability and QOL were also expected. Within at least 6 months of follow‐up, the current patient achieved these treatment goals without a severe adverse effect (Figs. 2 and 4; Table 1). The duration of treatment with AA among mild cases with adult HPP should be a subject for discussion in the future.

More than 400 pathogenic variants in the *ALPL* gene have been identified (https://alplmutationdatabase.jku.at/), and the residual TNSALP activity depends on the variant, zygosity, and whether a variant has a dominant negative effect. The current study identified a new pathogenic missense variant (c.319G > A; p.Val107Ile) with the result of functional analysis, showing that the identified variant in a homozygous manner had substantial enzymatic activity of the wild type (Fig. 3*B*). The residual enzymatic activity of this variant was much greater than that of the most prevalent pathogenic variant in the Japanese cohort (c.1559delT; p.Leu520ArgfsX86),^[^ ^26^ ^]^ which leads to the severe form of HPP in a homozygous fashion. The genetic and functional results were consistent with the typical and mild late‐onset clinical manifestations (permanent tooth loss and incomplete bilateral fractures in the diaphyses of femurs). Because the symptoms of patients with adult HPP vary widely from patient to patient, importantly, genetic testing and functional analysis of novel variants are indispensable for the diagnosis of HPP and consideration of AA initiation.

In the current case report, the potential negative effect of antiresorptive agents on a patient with HPP should be noted. In the condition of low bone formation rates, as represented by HPP and hypophosphatemic osteomalacia, antiresorptive drugs, including bisphosphonates and denosumab, which further lower bone turnover, are not recommended to avoid the development of undesirable pseudofractures, including atypical fractures.^[^ ^9^, ^11^ ^]^ Romosozumab, a monoclonal anti‐sclerostin antibody, is generally classified as an anabolic agent. However, romosozumab has some antiresorptive effects,^[^ ^27^ ^]^ and the possible contribution of pretreatment with romosozumab in the development of pseudofractures in the bilateral femoral diaphyses cannot be excluded. Although we cannot deny the negative effect of romosozumab on the development of pseudofractures in patients with HPP, romosozumab predominantly exerts an anabolic effect on the bone, and a recent article presented a potential benefit in patients with HPP.^[^ ^28^ ^]^ Therefore, the actual effect of romosozumab on the development of pseudofractures in patients with low bone formation rates, including HPP, warrants exploration. In addition, to avoid the use of antiresorptive drugs in patients with low bone formation rates, such as HPP, screening for serum ALP activity in addition to phosphate and calcium in osteoporotic patients is highly recommended before the initiation of antiresorptive drugs. A recent observational study demonstrated that 0.3% (5/1839) of the patients attending an osteoporotic clinic were diagnosed with HPP based on screening the level of ALP activity.^[^ ^29^ ^]^

There were some limitations in this study. First, this is a case report, and further studies including healthy people, patients with chronic kidney disease, or a larger number of patients with HPP are required to address whether uPPi/Cre could be an alternative marker to plasma PPi. Second, the follow‐up period was 6 months, which is insufficient to observe changes in pseudofractures. Last, plasma PLP was not measured because the measurement of PLP is not commercially available in Japan.

In summary, the current report on a patient with adult HPP revealed that treatment for an adult HPP patient with AA was effective for pseudofracture healing and improvement of physical function and QOL. Levels of uPPi/Cre decreased in parallel with plasma PPi concentration, suggesting that uPPi/Cre would be a convenient substitute for plasma PPi, which requires a complicated filtering process after blood sampling, for monitoring patients with HPP treated with AA. Moreover, a novel variant that causes HPP was identified by in vitro functional analysis. Adult HPP is a mild form of HPP in general; therefore, supportive care for arthritis and muscle pain and avoiding excessive physical training and the use of antiresorptive agents might be sufficient for most patients. However, as shown in the current case, enzyme replacement therapy facilitated fracture healing in a patient with adult HPP. Future studies must focus on the appropriate selection of patients with adult HPP for treatment with AA and exploration of adequate treatment duration with AA among these patients.

## Author Contributions

**Naoko Hidaka:** Conceptualization; data curation; formal analysis; writing – original draft. **Hiroaki Murata:** Data curation; writing – review and editing. **Kanako Tachikawa:** Formal analysis; methodology. **Keiichi Osaki:** Data curation. **Takashi Sekiyama:** Data curation. **Yuka Kinoshita:** Writing – review and editing. **Hajime Kato:** Conceptualization; writing – review and editing. **Yoshitomo Hoshino:** Writing – review and editing. **Soichiro Kimura:** Writing – review and editing. **Takashi Sunouchi:** Writing – review and editing. **So Watanabe:** Writing – review and editing. **Masaomi Nangaku:** Writing – review and editing. **Noriko Makita:** Writing – review and editing. **Toshimi Michigami:** Formal analysis; writing – review and editing. **Nobuaki Ito:** Conceptualization; supervision; writing – review and editing.

## Disclosures

NI has grant funding from Alexion Pharmaceuticals. All other authors state that they have no conflicts of interest.

## Patient Consent Statement

Written informed consent was obtained from the patient before the study.

### Peer Review

The peer review history for this article is available at https://www.webofscience.com/api/gateway/wos/peer‐review/10.1002/jbm4.10842.

## Supporting information


**Supplementary Figure S1.** Changes in serum alkaline phosphatase (ALP) and serum albumin‐corrected calcium (Ca) concentration after the initiation of asfotase alfa.

**Supplementary Table S1.** The scores of in silico analysis tools examining the currently identified variant in the *ALPL* gene (c.319G>A; p.Val107Ile).

## Data Availability

Not applicable.
