# Supplementary figures and images for: The Effect of Asfotase Alfa on Plasma and Urine Pyrophosphate Levels and Pseudofractures in a Patient With Adult‐Onset Hypophosphatasia
Source: JBMR Plus. 2023 Nov 20;7(12):e10842. doi: 10.1002/jbm4.10842 (PMC10731098; doi:10.1002/jbm4.10842)

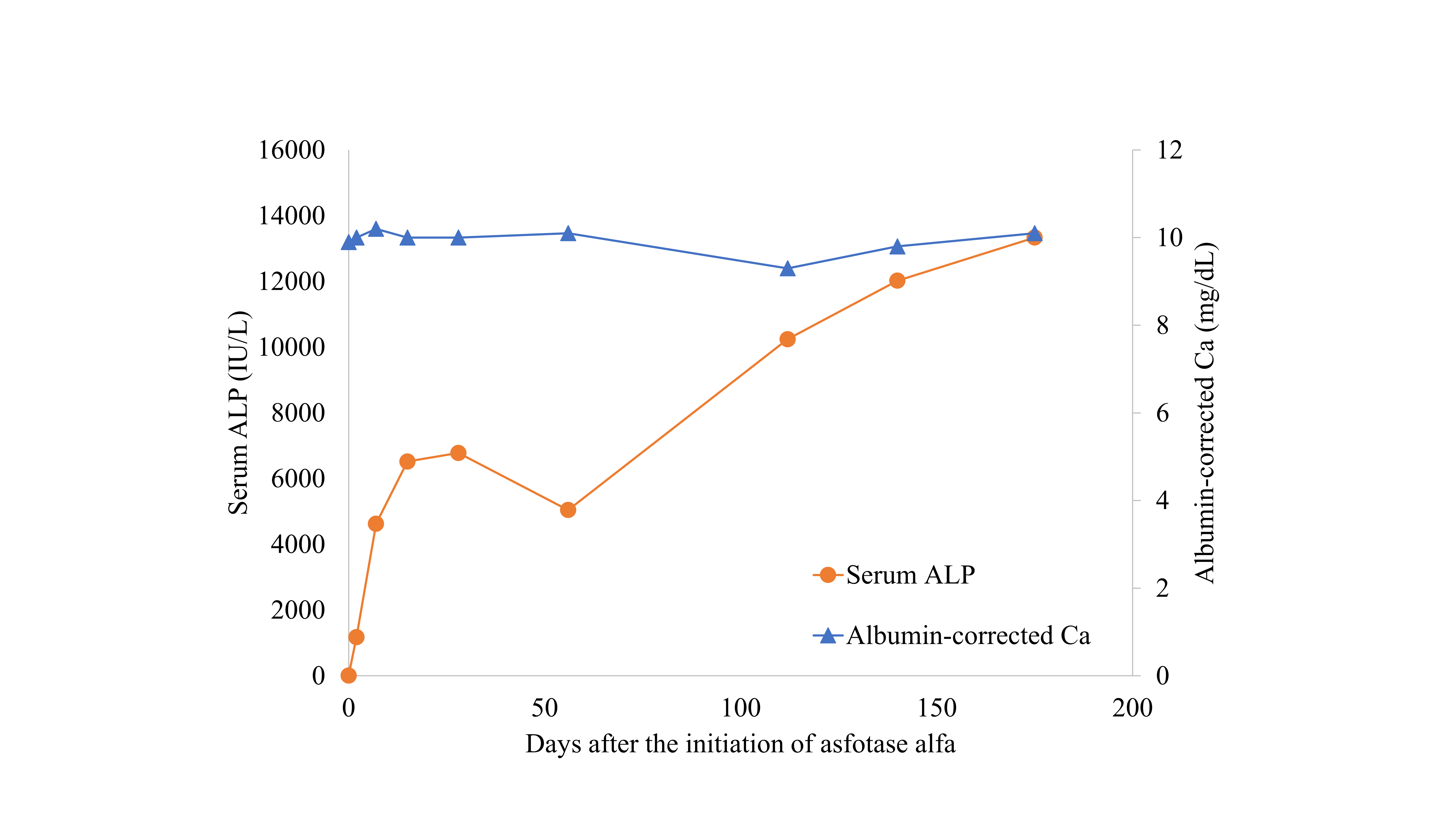

Supplement: Supplementary file 1 — Supplementary Figure S1. Changes in serum alkaline phosphatase (ALP) and serum albumin‐corrected calcium (Ca) concentration after the initiation of asfotase alfa. [file JBM4-7-e10842-s001.tiff]
