# Supplementary material for: The Effect of Asfotase Alfa on Plasma and Urine Pyrophosphate Levels and Pseudofractures in a Patient With Adult‐Onset Hypophosphatasia
Source: JBMR Plus. 2023 Nov 20;7(12):e10842. doi: 10.1002/jbm4.10842 (PMC10731098; doi:10.1002/jbm4.10842)
Supplement: Supplementary file 2 — Supplementary Table S1. The scores of in silico analysis tools examining the currently identified variant in the ALPL gene (c.319G>A; p.Val107Ile). [file JBM4-7-e10842-s002.docx]

**Supplemental Table 1**. The scores of *in silico* analysis tools examining the currently identified variant in the *ALPL* gene (c.319G>A; p.Val107Ile).

|  | SIFT ^a^ | Polyphen-2 ^b^ | CADD ^c^ | MutationTaster2021 ^d^ |
| --- | --- | --- | --- | --- |
| Score | 0.08 | 0.990 | 24.5 | Not provided |
| Prediction | Tolerated | Probably damaging | Deleterious ^e^ | Deleterious |

^a^ http://sift-dna.org/sift4g (1)

^b^ http://genetics.bwh.harvard.edu/pph2/ (2)

^c^ https://cadd.gs.washington.edu/ (3)

^d^ https://www.genecascade.org/MutationTaster2021/ (4)

^e^ Phred-like scaled CADD-score greater than 20 indicates the mutation is within the 1% most deleterious substitutions that we can do to the human genome. (3)

**References**

1. Vaser R, Adusumalli S, Leng SN, Sikic M, Ng PC：SIFT missense predictions for genomes. Nat Protoc 2015 111. 2015;11(1):1–9.

2. Adzhubei IA, Schmidt S, Peshkin L, et al.：A method and server for predicting damaging missense mutations. Nat Methods. 2010;7(4):248–9.

3. Rentzsch P, Schubach M, Shendure J, Kircher M：CADD-Splice-improving genome-wide variant effect prediction using deep learning-derived splice scores. Genome Med. 2021;13(1):31.

4. Steinhaus R, Proft S, Schuelke M, et al.：MutationTaster2021. Nucleic Acids Res. 2021;49(W1):W446–51.
